# Flexible modulation of hybrid feedback loops in competitive biological oscillators

**DOI:** 10.1038/s41540-025-00594-y

**Published:** 2025-11-03

**Authors:** Peng Zhao, Jian Liu, Tengfei Bao, Hong Huo, Ye Yuan, Tao Fang

**Affiliations:** 1 Department of Automation, Shanghai Jiao Tong University, Shanghai, China; 2 Institute of Machine Intelligence, University of Shanghai for Science and Technology, Shanghai, China

**Keywords:** Biophysics, Engineering, Neuroscience, Physics

## Abstract

Biological oscillators control vital rhythmic processes, and their dysregulation is associated with disorders such as cancer, sleep disturbances, and motor deficits. These oscillators often exhibit competitive interactions through mutual inhibition, and their dynamics are regulated by feedback mechanisms: positive feedback enhances synchronization, while negative feedback ensures tunability. However, the role of hybrid (positive-plus-negative) feedback in modulating competitive biological oscillators remains poorly understood. Here, we analyse seven competitive oscillators and demonstrate that hybrid feedback induces two distinct modulation modes: higher-amplitude, lower-frequency oscillations or higher-frequency, lower-amplitude oscillations, depending on hybrid feedback strengths. Furthermore, we show that oscillation tunability hinges on the asymmetry between positive and negative feedback loops. These findings deepen our understanding of oscillation regulation and could guide therapeutic strategies for diseases related to rhythm disorders.

## Introduction

Rhythmic behaviors rely on properly functioning biological oscillators, and their dysregulation can lead to severe disorders, some of which are life-threatening^1–5^. Many of these oscillators operate through mutual inhibition, forming competitive oscillators that ensure stable rhythmic activity^6–10^. For example, tightly regulated gene expressions, which are critical for organismal development and cellular homeostasis, often involve transcriptional oscillators that are modulated by mutually inhibited gene expressions. Disruptions in these competitive oscillators are implicated in diseases such as cancer^11,12^. Similarly, neurons exhibit intrinsic oscillations, and neural circuits, which are governed by mutual inhibition, regulate vital behaviors like sleep and locomotion. Dysfunction in these competitive oscillators can result in sleep disorders, seizures, or motor deficits^13,14^. Thus, competitive oscillators play fundamental roles in maintaining proper physiological function.

Biological oscillators are often regulated by feedback loops, which play essential roles in their function. Positive feedback reinforces signals^15^, enhances synchronization^16^, whereas negative feedback promotes oscillation generation^17,18^, ensures robustness^19^ in most oscillatory biological behaviors. For example, in the mammalian circadian clock, RORa and RORb act as positive regulators, driving *Bmal1* expression in the suprachiasmatic nuclei^15^. When biological oscillators are coupled with each other, synchronization is facilitated through positive feedback interactions^16^. In contrast, time-delayed negative feedback in protein synthesis can induce periodic oscillations^18^. Synthetic circuits also demonstrate that negative feedback enhances oscillator robustness^19^. The integration of both feedback types further fine-tunes dynamical control in biological oscillators^20–22^. For instance, combined positive and negative feedback not only improves noise resistance but also increases the tunability of key oscillation parameters^22^.

Competitive biological oscillators governed by mutual inhibition are dynamically modulated by both positive and negative feedback, giving rise to diverse rhythmic behaviors. Three primary circuit architectures have been demonstrated to modulate competitive oscillators: (i) purely positive feedback^23–25^, (ii) purely negative feedback^26,27^, and (iii) hybrid(positive-plus-negative) feedback^9,10,28–32^. For instance, in cell cycle regulation, the activation and inactivation of Cdk1 during mitotic entry and exit involve a positive feedback loop with Cdc25 and Wee1, forming a bistable trigger essential for cell cycle progression^23^. Conversely, in protein stress responses, AMPK, ULK1, and MTORC1 interact through negative feedback, generating high-amplitude oscillations in AMBRA1 phosphorylation under mild stress^26^. Hybrid feedback, however, is particularly widespread, appearing in key biological processes such as the cell cycle^32^, neuronal networks^29^, circadian rhythms^30^, gene transcriptional regulation^28^, predator-prey systems^31^, brain activity^10^, and locomotor control^9^. In the mammalian cell cycle, hybrid feedback precisely orchestrates the antagonistic relationship between CycB and Cdh1 to drive phase transitions^32^. Neuronal networks leverage hybrid feedback to create stable toggle switches, where mutual inhibition coupled with regulatory loops enables bifurcation-controlled state transitions^29^. Circadian clocks integrate multiple feedback loops, with Per2, Cry1, and Rev-erb-α forming a mutually repressive core that sustains endogenous oscillations^30^. Gene regulatory networks exploit interlinked feedback motifs to generate tuneable dynamics, functioning as bistable switches, oscillators, or excitable systems^28^. Ecological models reveal that hybrid feedback in predator-prey systems, combining parasitism and mutualism, shapes ecosystem stability and diversification^31^. In cortical microcircuits, excitatory-inhibitory interactions implement rate- and oscillation-based computations, producing synchronized state transitions across cortical layers^10^. Even locomotor control in *Drosophila* larvae arises from mechanical feedback loops, where interacting spring-like forces generate coordinated waves, oscillations, and chaotic deformations^9^. The widespread occurrence of hybrid feedback across biological scales suggests an evolutionary optimization for tuneable, robust oscillations. Yet, despite its prevalence, the precise mechanisms by which hybrid feedback shapes rhythmic patterns in competitive oscillators remain incompletely resolved, highlighting a critical gap in our understanding of biological dynamics.

Here, we investigate how hybrid feedback modulates rhythmic dynamics in competitive oscillators across the above-mentioned seven classes of biological processes or systems. Analyzing models ranging from mammalian cell cycles to six additional biological oscillators, we discover two distinct modulation modes: higher-amplitude, lower-frequency oscillations or higher-frequency, lower-amplitude oscillations, depending on hybrid feedback strengths. Furthermore, we demonstrate that oscillation tunability scales with the asymmetry between positive and negative feedback loops, highlighting a key design principle for biological rhythm regulation. These findings provide new insights into oscillation regulation mechanisms and could guide therapeutic strategies for diseases related to rhythm disorders.

## Results

### Hybrid feedback regulation in mammalian cell cycle competitive oscillators

**Fig. 1:**
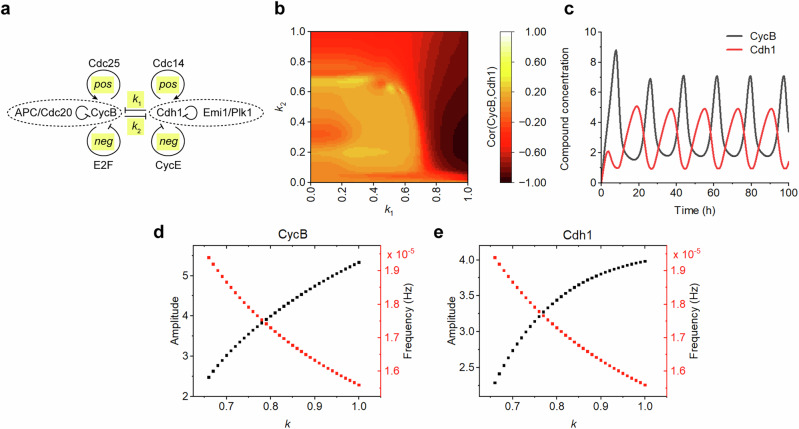
Competitive dynamics between CycB and Cdh1 in the mammalian cell cycle. **a** Schematic representation of key regulatory interactions in the mammalian cell cycle. **b** Correlation heatmap showing phase relationship between CycB and Cdh1 across different mutual inhibition strengths (*k*_1_ and *k*_2_). **c** Time-course oscillations of CycB and Cdh1 protein levels under strong mutual inhibition (*k*_1_ = *k*_2_ = 1). Quantitative analysis of oscillation properties: **d** CycB and **e** Cdh1 amplitude and frequency as functions of competitive strength *k*, demonstrating an inverse relationship between amplitude and frequency scaling.

To investigate how competitive interactions and feedback loops regulate oscillatory dynamics, we developed a computational model based on ordinary differential equations representing two core mammalian cell cycle oscillators, as outlined in the prior influence diagram^32^. CycB and Cdh1, which are essential for the mammalian cell cycle, exhibit mutually inhibitory behavior: in the G1 steady state, the level of CycB is low while Cdh1 is active, whereas in the M steady state, CycB is active while Cdh1 is inactive. CycB and Cdh1 are further modulated by additional regulatory compounds. To recapture these dynamics, our model incorporates: (i) two primary negative feedback loops, [CycB → APC/Cdc20 ⊣ CycB] and [Cdh1 ⊣ Plk1 ⊣ Emi1 ⊣ Cdh1], as core oscillators; (ii) two positive feedback loops, [CycB → Cdc25 → CycB] and [Cdh1 → Cdc14 → Cdh1], for additional regulation; (iii) two secondary negative feedback loops, [CycB ⊣ E2F → CycB] and [Cdh1 → CycE ⊣ Cdh1], for modulation (Fig. 1a). The model effectively recaptures the antagonistic relationship between CycB and Cdh1, which are further fine-tuned by two positive-feedback and two negative-feedback loops, respectively.

We quantified the mutual inhibition between CycB and Cdh1, assigning strength parameters *k*_1_ (Cdh1 inhibits CycB) and *k*_2_ (CycB inhibits Cdh1), while additional positive and negative feedback effects were represented by *pos* and *neg*, respectively. Initial simulations focused exclusively on competitive interactions by setting *neg* = 0 and *pos* = 0, with other parameters maintained at values known to produce stable oscillations from previous studies^6,22,32^. Systematically increasing *k*_1_ and *k*_2_ initially failed to produce correlated dynamics, with robust antiphase oscillations emerging only at higher inhibition strengths (Fig. 1b, c). To quantitatively assess how competition strength affects oscillator properties, we set *k*_1_ = *k*_2_ = *k* (where *k* ∈ [0,1]) and measured the resulting amplitude and frequency of these antiphase oscillations. Our analysis revealed that competition strength inversely modulates oscillatory properties: stronger competition (higher *k* values) led to increased oscillation amplitudes but decreased frequencies in both CycB and Cdh1 (Fig. 1d, e). These findings highlight how mutual inhibition tunes the trade-off between amplitude and frequency in coupled oscillators.

**Fig. 2:**
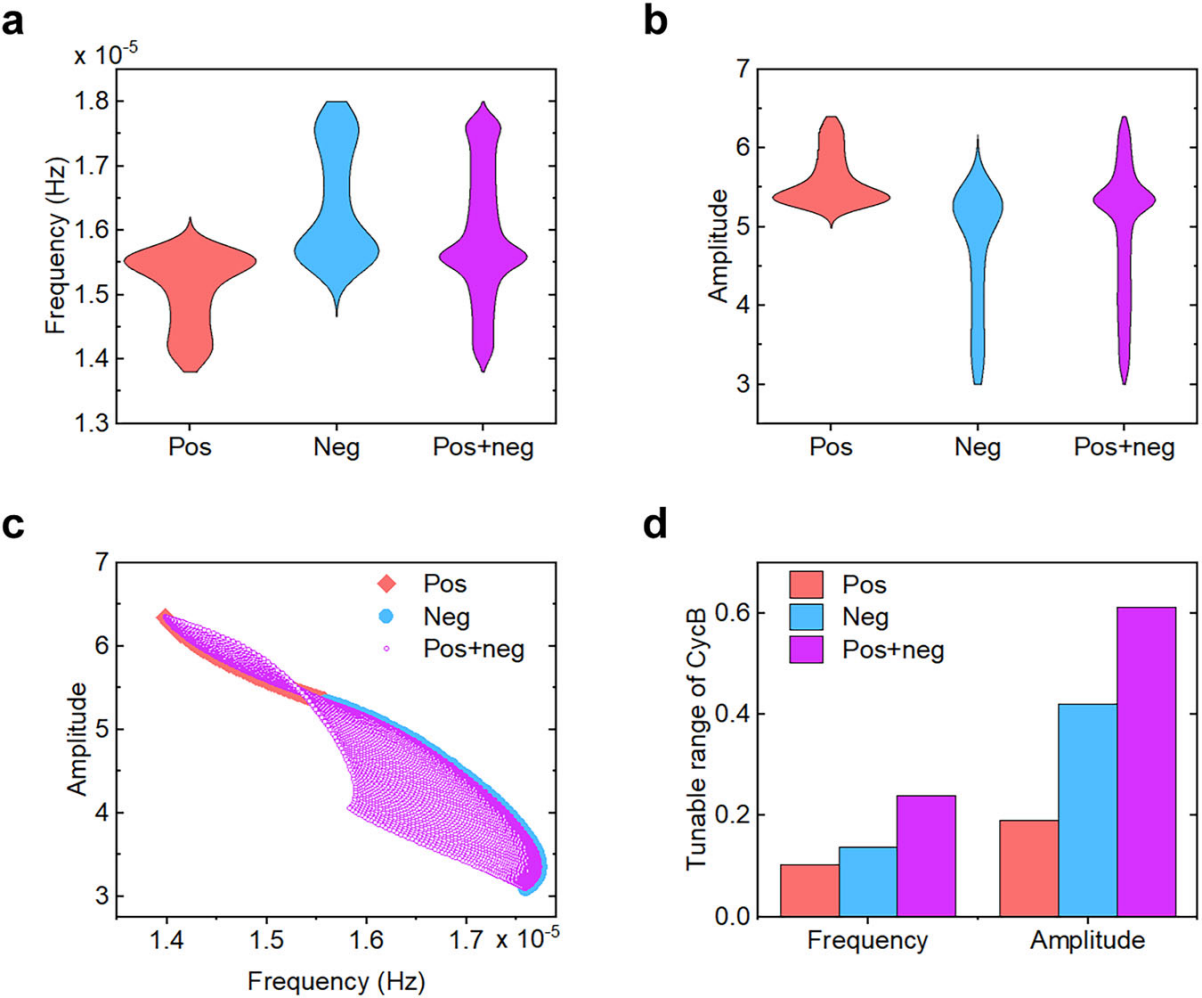
Modulation of CycB oscillation properties by feedback loops. **a** Frequency distributions for three feedback configurations: positive-only (*neg* = 0), negative-only (*pos* = 0), and hybrid (positive-plus-negative) feedback. **b** Corresponding amplitude distributions, with violin plot width representing density. **c** The amplitude/frequency scatters across all feedback conditions. **d** Normalized tunability ranges for CycB frequency (left) and amplitude (right) under each feedback configuration, calculated by (maximum - minimum)/reference value, where the reference value is the baseline (*pos* = 0, *neg* = 0) of frequency/amplitude.

To investigate how supplementary feedback loops modulate CycB and Cdh1 dynamics, we systematically varied positive (*pos*) and negative (*neg*) feedback strengths across the range [0,1], while maintaining constant competition strength (*k*_1_ = *k*_2_ = 1) to preserve antiphase oscillations. Our analysis revealed two distinct regulatory patterns: (i) in single-feedback versions, negative feedback alone (*pos* = 0) generated higher-frequency oscillations compared to positive feedback alone (*neg* = 0), whereas positive feedback alone yielded larger amplitudes than negative feedback alone; (ii) in the hybrid-feedback version, combining positive and negative feedback recapitulated the full range of frequencies and amplitudes as seen in single-feedback systems (Fig. 2a, b), yet also exhibited additional properties. Scatter plot analysis revealed superior tunability beyond a simple additive effect of individual feedback loops (Fig. 2c), with CycB oscillation displaying particularly enhanced control over both frequency and amplitude (Fig. 2d). Cdh1 oscillations exhibited analogous modulation patterns with CycB (Supplementary Fig. 1), confirming consistent regulatory effects across competitive oscillators. Thus, hybrid feedback enables dual modulation, permitting higher-amplitude, lower-frequency oscillations and higher-frequency, lower-amplitude oscillations, while significantly expanding the tuneable parameter space in both dimensions.

### **Hybrid feedback regulation in six additional competitive oscillator models**

To evaluate the generalizability of enhanced tunability through hybrid feedback in competitive oscillators, we analyzed six additional biological oscillator models: (i) Fitzhugh-Nagumo, a well-studied model in neuronal networks^29,33,34^, (ii) Goodwin, an oscillator relevant to circadian rhythms^35–39^, (iii) Repressilator, a tripe-negative feedback loop in transcriptional regulation^40,41^, (iv) Predator-prey, a model employed in ecological systems^42–44^, (v) Van der Pol, a model could describe brain activity^10,45,46^, (vi) Neuromechanical oscillator, a model proposed for motor control^47,48^. These models represent diverse biological systems where competitive oscillators naturally arise, often incorporating additional feedback loops to fine-tune their dynamics. In the neuronal networks, for example, neurons frequently couple through mutual repression and are embedded in motifs containing both positive and negative feedback loops^29^. Similarly, in the plant circadian clock, the mutually inhibited genes LHY and TOC1, which are modulated by additional feedback loops, regulate the rhythm at the morning phase and evening phase, respectively^39^. Gene regulatory networks also commonly feature mutually inhibited transcription factors under the influence of supplementary feedback loops^41^. Ecological systems exhibit analogous dynamics, where predator populations may compete through mutual inhibition while also engaging in mutualism and parasitism^44^. In brain regions, PV1 and PV2 exhibit mutual inhibition with additional feedback modulation^10^. In motor control, muscles often show mutual inhibition because of structural constraints, further regulated by neuron-muscle or muscle-muscle feedback^47^.

To systematically compare these systems, we incorporated competitive interactions (mutual inhibition) and additional positive and negative feedback loops (Supplementary Fig. 2). To establish a consistent baseline, we initialized each system with high mutual inhibition strengths (*k*_1_, *k*_2_) to guarantee antiphase oscillations. Specific strengths for the positive (*pos*) and negative (*neg*) feedback loops were then applied. The transient and equilibrium dynamics, visualized through phase portraits and time-series plots (Supplementary Fig. 3), reveal the core behavior of each system. Furthermore, we assessed the robustness of these dynamics by conducting parameter sweeps of the *pos* and *neg* values (Supplementary Fig. 4). This analysis consistently showed that higher positive feedback strength increases amplitude while decreasing frequency, whereas negative feedback strength produces the inverse effect.

**Fig. 3:**
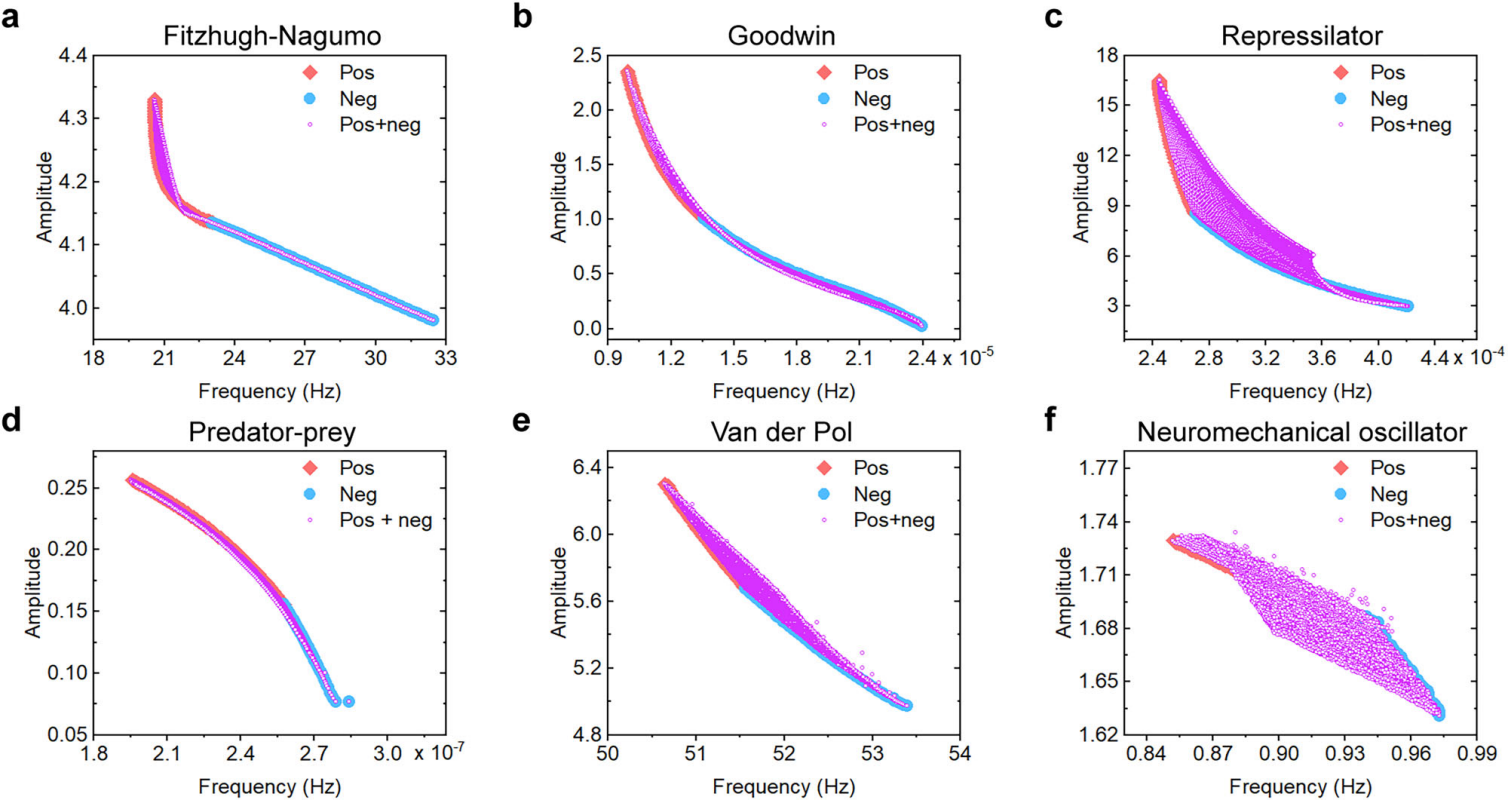
Amplitude-frequency relationships of oscillation properties across six competitive biological models. **a** Fitzhugh-Nagumo model (neuronal activity). **b** Goodwin oscillator (circadian rhythm). **c** Repressilator (genetic transcriptional network). **d** Predator-prey system (ecological dynamics). **e** Van der Pol oscillator (brain region activity). **f** Neuromechanical oscillator (motor control).

**Fig. 4:**
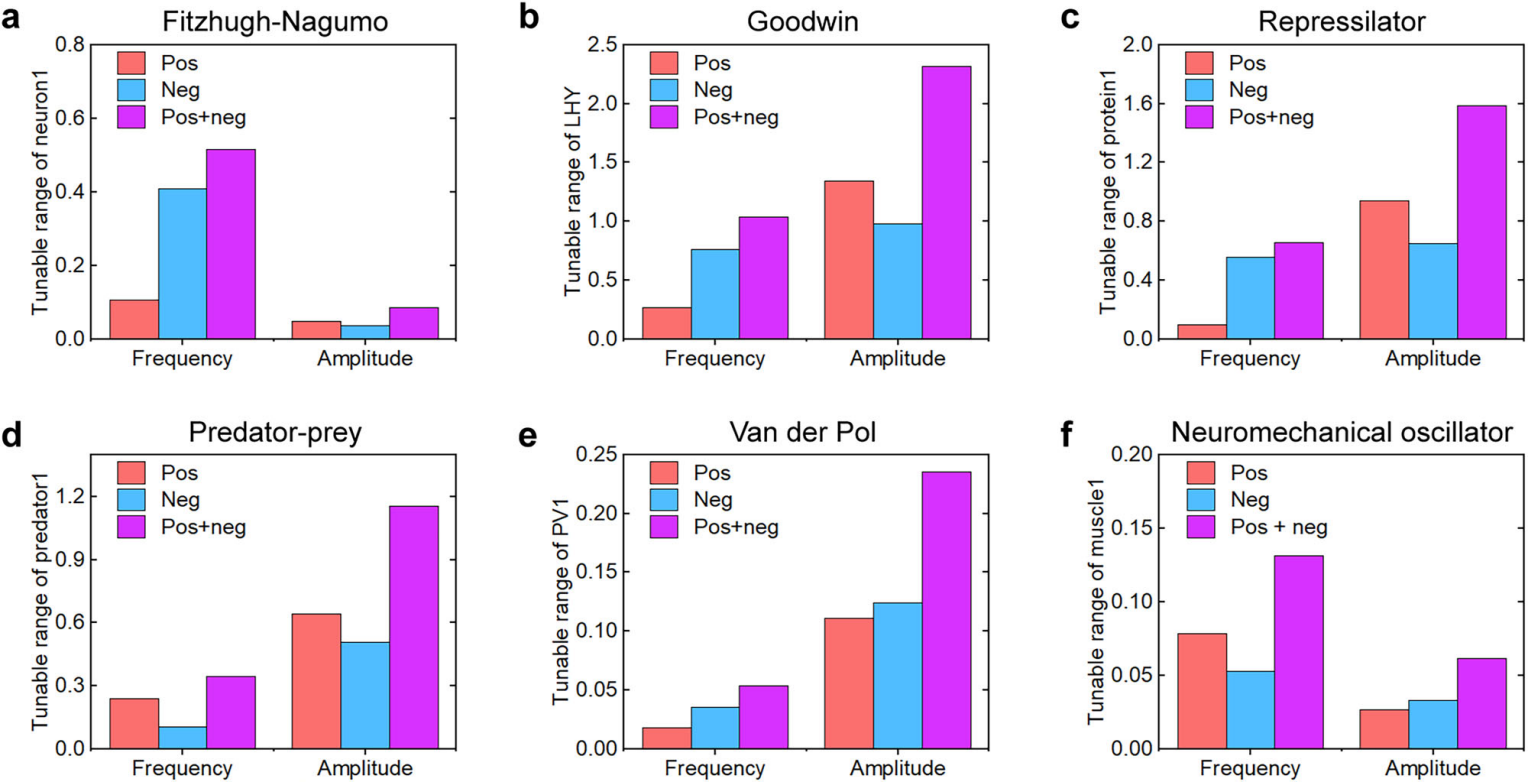
Comparative analysis of oscillation tunability ranges. Normalized tunability ranges for frequency (left) and amplitude (right) across six competitive biological models: **a** Fitzhugh-Nagumo. **b** Goodwin. **c** Repressilator. **d** Predator-prey. **e** Van der Pol. **f** Neuromechanical oscillator. Tunability is calculated as (maximum - minimum)/reference value, where reference represents the no-feedback condition (*pos* = 0, *neg* = 0).

For all models, we quantified oscillation properties (amplitude and frequency) under three feedback configurations: positive feedback only, negative feedback only, and hybrid feedback. This systematic approach tests whether hybrid feedback universally enhances tunability across competitive biological oscillators. The amplitude-frequency scatter plots revealed consistent patterns across models: positive feedback alone generated low-frequency, high-amplitude oscillations, whereas negative feedback alone produced high-frequency, low-amplitude oscillations, with hybrid feedback spanning the full range between these extremes (Fig. 3). Given that biological oscillators operate across periods ranging from seconds to days, resulting in substantial frequency variation, while amplitudes remained relatively stable, except in the Repressilator, where amplitude varied nearly eight folds (Fig. 3c). Further analysis of three feedback configurations demonstrated distinct tuning capabilities across six oscillator models. Although positive and negative feedback differentially modulated the frequency and amplitude, hybrid feedback consistently exhibited broader normalized tuning ranges for both frequency and amplitude compared to single-feedback systems (Fig. 4). Thus, hybrid feedback offered two key advantages: (i) expanded coverage of amplitude-frequency combinations beyond the simple additive effects of single-feedback modes, and (ii) significantly enhanced tunability in both frequency and amplitude compared to single-feedback configurations.

### Enhanced tunability through asymmetric feedback loops

To investigate how feedback loop asymmetry affects oscillation tunability, we systematically varied the relative strengths of positive and negative feedback while maintaining antiphase oscillations in competitive oscillators. Our analysis revealed that hybrid feedback modulates oscillations through mechanisms distinct from the simple superposition of individual feedback effects. We further examined whether the relative strength of positive-to-negative or negative-to-positive feedback influences these additional tuned domains. By varying the feedback ratios across seven biological oscillator models, we calculated the frequency and amplitude for each condition and quantified the proportion of extra-tuned domains relative to all achievable amplitude-frequency ranges under hybrid feedback.

**Fig. 5:**
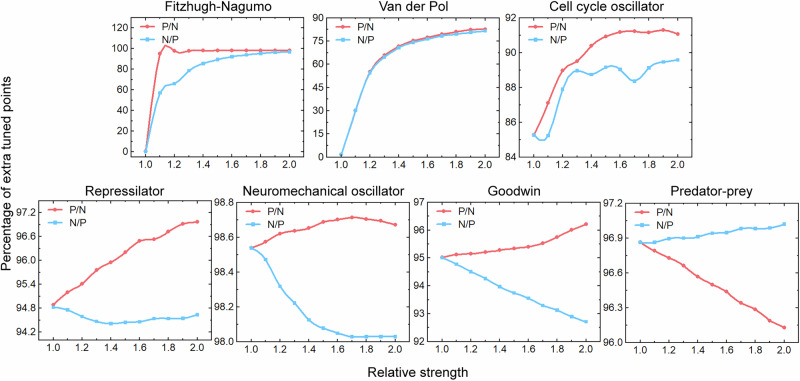
Enhanced tunability through asymmetrical feedback loops in competitive biological oscillators. Percentage of additional accessible states (vertical axis) as a function of relative strength (horizontal axis) of feedback loops for seven competitive oscillators. The percentage of additional states was calculated as (*N*_*h*_ – *N*_*s*_)/*N*_*h*_ × 100%, where *N*_*h*_ and *N*_*s*_ represent the number of accessible states under hybrid feedback and single feedback, respectively.

Our initial experiments, conducted using the Fitzhugh-Nagumo and Van der Pol models for their symmetrical feedback structures, revealed that imbalanced feedback strengths, whether positive- or negative-dominant, significantly expanded the accessible tuning domains (Fig. 5). In the Fitzhugh-Nagumo model, the proportion of extra tuned domains surged from near zero to almost 100% under positive-dominant conditions and to near 60% under negative-dominant conditions as the relative strength of the dominant feedback increased, eventually plateauing in both regimes. The Van der Pol model exhibited a sharp initial increase from zero to 60%, followed by slower growth. Extending this analysis to five additional models with distinct feedback structures consistently demonstrated enhanced tunability under asymmetric feedback conditions (Fig. 5). Specifically, increasing the relative strength of positive-to-negative feedback boosted tunability in the cell cycle oscillator (from ~85.5% to ~91%), the Repressilator (from ~94.8% to ~97%), the Neuromechanical oscillator (from ~98.55% to ~98.7%), and the Goodwin model (from ~95% to ~96.5%). Conversely, increasing negative-to-positive feedback initially reduced tunability in the cell cycle oscillator (to ~85%) before a rise to 89%, and produced declines in the Neuromechanical oscillator (to ~98%), the Goodwin model (to ~92.5%), and the Repressilator (to ~94.5%), followed by a slight recovery in the latter. In contrast, the Predator-prey model was dominated by negative feedback, with tunability increasing alongside negative-to-positive feedback strength (from ~96.9% to ~97.1%) and decreasing with enhanced positive feedback (to ~96.1%). Together, these findings demonstrate that while a balance exists between the two feedback types, moving beyond this equilibrium toward either form of asymmetry generally enhances tunability across a wide range of competitive oscillatory models.

## Discussion

Our study elucidates a fundamental design principle governing biological rhythmicity: hybrid feedback, when coupled with competitive mutual inhibition, dramatically expands the tunability of oscillatory dynamics. We demonstrated this across seven distinct biological oscillator models, revealing that the relative asymmetry between feedback strengths is a key determinant of the accessible amplitude-frequency space. This enhanced tunability provides a versatile mechanism for biological systems to generate a wide repertoire of rhythms from a core competitive circuit, a capability crucial for adapting to internal and external demands.

A foundational observation from our analysis of the mammalian cell cycle model was that increasing the strength of mutual inhibition (parameter *k*) led to an increase in oscillation amplitude but a concurrent decrease in frequency. This inverse relationship can be understood through the lens of nonlinear dynamics. Stronger mutual inhibition deepens the potential wells defining the system’s stable states (e.g., G1 and M phases), increasing the energy barrier between them. Transitions between these states, driven by the slower, integrating action of the supplementary feedback loops, thus become less frequent (lower frequency) but more decisive, resulting in sharper, more pronounced switches (higher amplitude). This trade-off establishes a baseline dynamic range upon which additional feedback acts.

The core of our findings lies in the superior performance of hybrid feedback over purely positive or negative configurations. The mathematical principle behind this is the introduction of multi-timescale dynamics and nonlinear interactions. Positive feedback typically acts as an accelerant, promoting rapid, all-or-nothing transitions that favor high-amplitude outputs but can lead to bistability rather than oscillation if unchecked^22^. Negative feedback provides the necessary restorative force to create a stable limit cycle, often leading to faster, smaller-amplitude oscillations^28^. When combined, they do not merely act additively. Their interaction can create complex nullclines and bifurcation structures. This complexity manifests as a broader range of stable oscillatory regimes, allowing the system to access amplitude-frequency combinations that are unreachable by either feedback type alone. This explains the “extra tuned domains” we observed in the scatter plots, which represent novel dynamic states emergent from the nonlinear coupling of the two feedback types.

A particularly significant finding is that this enhanced tunability is maximized under conditions of asymmetric feedback strength. This phenomenon, prominently observed in the Fitzhugh-Nagumo and Van der Pol models, can be explained by breaking the symmetry of the system’s phase space. In symmetric or balanced feedback conditions, the system’s dynamics are often constrained to a narrower corridor of possible states. Introducing asymmetry, whether positive- or negative-dominant, effectively warps the phase space, creating new basins of attraction and altering the shape and location of the limit cycle. This expands the envelope of achievable amplitudes and frequencies. Biologically, this suggests that evolvable oscillatory circuits are not necessarily optimized for balanced feedback but rather for a tuneable imbalance, allowing one type of feedback to be preferentially recruited to shift rhythms between a high-amplitude/low-frequency state and a low-amplitude/high-frequency state.

Our comparative analysis across diverse models, Fitzhugh-Nagumo, Van der Pol, Repressilator, Predator-Prey, Goodwin, and the Neuromechanical oscillator, confirms that this is a general principle, though its specific manifestation depends on the intrinsic dynamics of each system. For instance, the Repressilator’s core triple-negative-feedback and Goodwin’s negative-feedback designs are inherently oscillatory but often produce low-amplitude oscillations^35,36,40,41^. Our results show that incorporating asymmetric hybrid feedback can increase its amplitude range, making it a far more robust and tuneable genetic clock. Conversely, the Predator-Prey model, which is naturally dominated by negative feedback, namely consumption and mortality^42–44^, showed its greatest tunability when this negative dominance was reinforced. This indicates that the universal applicability of asymmetric feedback lies not in a single recipe that always favors positive feedback, but in the general strategy of breaking symmetry to access a wider parameter space. The specific optimal asymmetry is a property of the underlying oscillator’s structure.

An important factor not explicitly explored in our current models but critical to biological realism is time delay. In many of the systems studied, from transcriptional feedback in the Repressilator and Goodwin models to Neuromechanical signaling, feedback loops are not instantaneous^35,36,40,41^. Time delays in negative feedback are a classic mechanism for generating oscillations, as they can cause an overshoot in the system’s response to a change. In a hybrid setup, delays can introduce crucial phase lags between the positive and negative signals. For example, in the Neuromechanical oscillator, a delay in the sensory feedback from a muscle’s stretch could ensure that excitatory (positive) feedback peaks just as the muscle is at its optimal length for contraction, while delayed inhibitory (negative) feedback arrives to terminate the contraction and initiate the antagonist’s movement^47,48^. This orchestration of phase via delay could be a key mechanism for precisely tuning both the frequency and waveform of the resulting rhythm, and its integration with hybrid feedback represents a vital avenue for future research.

While our study provides broad insights, several limitations must be acknowledged. First, our models are necessarily simplifications of a vastly more complex biological reality. We represent feedback with single strength parameters (*pos*, *neg*), whereas in vivo, these loops involve multi-step biochemical reactions with their own nonlinearities and delays. Second, we focused on deterministic models, whereas biological systems operate in a noisy environment. Another limitation is that our approach complements these summaries but relies solely on statistical metrics. Future work could investigate how stochastic fluctuations interact with hybrid feedback to affect the robustness and reliability of the tuned oscillations.

In conclusion, our work establishes asymmetric hybrid feedback as a powerful and general mechanism for enhancing the tunability of competitive biological oscillators. By providing a unified framework for understanding rhythm regulation across cellular, neural, genetic, and ecological domains, these findings offer a new perspective on the design principles of biological clocks. This knowledge could inform the development of therapeutic strategies for rhythm disorders by suggesting novel targets for manipulating feedback asymmetry to restore healthy dynamics, as well as guide the engineering of synthetic biological oscillators with precisely specified properties.

## Methods

We numerically solved the ordinary differential equations (ODEs) for each oscillator model using Runge-Kutta integration in MATLAB. For each model, we initialized parameters from published values known to produce stable limit cycle oscillations, and we determined the (*k*_1_, *k*_2_) parameter space, maintaining antiphase oscillations between competitive oscillators. We systematically varied positive (*pos*) and negative (neg) feedback strengths while computing limit cycle solutions and recording amplitude and frequency characteristics. Finally, we modulated the relative feedback strength ratio (N/P and P/N) to assess its impact on oscillation properties. The complete set of ODE formulations and parameter values used for each oscillator model is provided below.

### Cell cycle oscillator model

We constructed a mammalian cell cycle oscillator using a system of ten ODEs. As outlined in the prior influence diagram^32^, we abstract the core model includes two interlocked negative feedback loops that generate autonomous oscillations, mutual inhibition between the CycB and Cdh1 oscillators, and experimentally validated parameters that reproduce established oscillation patterns for CycB and Cdh1 activity. Parameter nomenclature follows established conventions. *k*_*s*_ is the synthesis rate. *k*_*a*_ is the activation rate. *k*_*i*_ is the inactivation rate. *k*_*d*_ is the degradation rate. The model also incorporates four additional regulatory elements that implement two positive-feedback loops and two negative-feedback loops (Fig. 1a), which interact to fine-tune the overall dynamics. Specifically, Cdc25 and E2F enhance CycB synthesis, while Cdc20 and Cdh1 promote degradation of CycB. Conversely, Cdc14 increases Cdh1 synthesis, while Emi1, CycE, and CycB accelerate the degradation of Cdh1. The model further depicts activation chains [CycB → APC → Cdc20] and inactivation chains [Cdh1 → Plk1 → Emi1]. Regulatory crosstalk is also captured; for instance, high CycB levels promote E2F degradation yet stimulate Cdc25 synthesis, while elevated Cdh1 levels inhibit CycE degradation and promote Cdc14 synthesis.

1 $$\frac{{\rm{d}}CycB}{{\rm{d}}t}=\frac{1}{{\tau }_{1}}\left(\begin{array}{c}{k}_{sCycB}-{k}_{dCycB}CycB+{k}_{aCycB}\frac{{({k}_{p}Cdc25)}^{n}}{{\theta }_{Cdc25}^{n}\,+{\,({k}_{p}Cdc25)}^{n}}+{k}_{aCycB}^{{\prime} }\frac{E2{F}^{n}}{{\theta }_{E2F}^{n}\,+\,E2{F}^{n}}\\ -\left({k}_{dCycB}^{{\prime} }\frac{Cdc{20}^{n}}{{\theta }_{Cdc{20}^{n}}\,+\,Cdc{20}^{n}}+{k}_{dCycB}^{{\prime} {\prime} }\frac{{({k}_{1}Cdh1)}^{n}}{{\theta }_{Cdh1}^{n}\,+\,{({k}_{1}Cdh1)}^{n}}\right)CycB\end{array}\right)$$

2 $$\frac{{\rm{d}}Cdh1}{{\rm{d}}t}=\frac{1}{{\tau }_{2}}\left(\begin{array}{l}{k}_{sCdh1}-{k}_{dCdh1}Cdh1+{k}_{aCdh1}\frac{{({k}_{p}Cdc14)}^{n}}{{\theta }_{Cdc14}^{n}\,+\,{({k}_{p}Cdc14)}^{n}}\\ -\left({k}_{dCdh1}^{{\prime} }\frac{Emi{1}^{n}}{{\theta }_{Emi1}^{n}\,+\,Emi{1}^{n}}+{k}_{dCdh1}^{{\prime} {\prime} }\frac{{({k}_{n}CycE)}^{n}}{{\theta }_{CycE}^{n}\,+\,{({k}_{n}CycE)}^{n}}+{k}_{iCdh1}\frac{{({k}_{2}CycB)}^{n}}{{\theta }_{CycB}^{n}\,+\,{({k}_{2}CycB)}^{n}}\right)Cdh1\end{array}\right)\,\,\,\,\,$$

3 $$\begin{array}{l}\frac{{\rm{d}}APC}{{\rm{d}}t}=\frac{1}{{\tau }_{1}}\left({k}_{aAPC}\frac{Cyc{B}^{n}}{{\theta }_{CycB}^{n}\,+\,Cyc{B}^{n}}\left(AP{C}_{tot}-APC\right)-{k}_{dAPC}APC\right)\end{array}$$

4 $$\begin{array}{l}\frac{{\rm{d}}Cdc20}{{\rm{d}}t}=\frac{1}{{\tau }_{1}}\left({k}_{aCdc20}\frac{AP{C}^{n}}{{\theta }_{APC}^{n}+AP{C}^{n}}\left(Cdc{20}_{tot}-Cdc20\right)-{k}_{dCdc20}Cdc20\right)\end{array}$$

5 $$\begin{array}{l}\frac{{\rm{d}}Plk1}{{\rm{d}}t}=\frac{1}{{\tau }_{2}}\left({k}_{sPlk1}-{k}_{dPlk1}Cdh1-{k}_{dCdh1}^{{\prime} }\frac{Cdh{1}^{n}}{{\theta }_{Cdh1}^{n}+Cdh{1}^{n}}Plk1\right)\end{array}$$

6 $$\begin{array}{l}\frac{{\rm{d}}Emi1}{{\rm{d}}t}=\frac{1}{{\tau }_{2}}\left({k}_{sEmi1}-{k}_{dEmi1}Emi1-{k}_{dEmi1}^{{\prime} }\frac{Plk{1}^{n}}{{\theta }_{Plk1}^{n}+Plk{1}^{n}}Emi1\right)\end{array}$$

7 $$\begin{array}{l}\frac{{\rm{d}}E2F}{{\rm{d}}t}=\frac{1}{{\tau }_{1}}\left({k}_{sE2F}-\left({k}_{dE2F}+{k}_{dE2F}^{{\prime} }\frac{{({k}_{n}CycB)}^{n}}{{\theta }_{CycB}^{n}+{({k}_{n}CycB)}^{n}}\right)E2F\right)\end{array}$$

8 $$\begin{array}{l}\frac{{\rm{d}}CycE}{{\rm{d}}t}=\frac{1}{{\tau }_{2}}\left({k}_{sCycE}-\left({k}_{dCycE}-{k}_{dCycE}^{{\prime} }\frac{Cdh{1}^{n}}{{\theta }_{Cdh1}^{n}+Cdh{1}^{n}}\right)CycE\right)\end{array}$$

9 $$\begin{array}{l}\frac{{\rm{d}}Cdc25}{{\rm{d}}t}=\frac{1}{{\tau }_{1}}\left({k}_{aCdc25}\frac{Cyc{B}^{n}}{{\theta }_{CycB}^{n}+Cyc{B}^{n}}\left(Cdc{25}_{tot}-Cdc25\right)-{k}_{dCdc25}Cdc25\right)\end{array}$$

10 $$\begin{array}{l}\frac{{\rm{d}}Cdc14}{{\rm{d}}t}=\frac{1}{{\tau }_{2}}\left({k}_{aCdc14}\frac{Cdh{1}^{n}}{{\theta }_{Cdh1}^{n}+Cdh{1}^{n}}\left(Cdc{14}_{tot}-Cdc14\right)-{k}_{dCdh1}Cdc14\right)\end{array}$$

Where the *CycB*, *Cdh*1, *APC*, *Cdc*20, *Plk*1, *Emi*1, *E*2*F*, *CycE*, *Cdc*25, and *Cdc*14 represent the concentration of compounds related to DNA synthesis in the mammalian cell cycle.

Initial conditions:

All variables start with random values in [0,1].

Parameters:

*τ*_1_ = 7, *τ*_2_ = 11.5, *k*_*n*_ = *neg*, *k*_*p*_ = *pos*, *k*_*sCycB*_ = 10, *k*_*aCycB*_ = 10, *k*_*dCycB*_ = 1, *k*^′^_*dCycB*_ = 10, *k*^″^_*dCycB*_ = 10, *k*^″^_*aCycB*_ = 10, *k*_*sCdh*1_ = 10, *k*_*aCdh*1_ = 10, *k*_*dCdh*1_ = 1, *k* = 10*, k* = 10, *k*_*iCdh*1_ = 10, *k*_*aAPC*_ = 1, *k*_*dAPC*_ = 1, *k*_*aCdc*20_ = 1, *k*_*dCdc*20_ = 1, *k*_*sPlk*1_ = 10, *k*_*dPlk*1_ = 1, *k* = 10, *k*_*sEmi*1_ = 10, *k*_*dEmi*1_ = 1, *k* = 10, *k*_*aCdc*25_ = 1, *k*_*dCdc*25_ = 1, *k*_*aCdc*14_ = 1, *k*_*dCdc*14_ = 1, *k*_*sE*2*F*_ = 10, *k*_*dE*2*F*_ = 1, *k* = 10, *k*_*sCycE*_ = 10, *k*_*dCycE*_ = 5, *k*^″^_*dCycE*_ = 5, *n* = 4, *θ*_*CycB*_ = 5, *θ*_*Cdh*1_ = 5, *θ*_*Cdc*25_ = 5, *θ*_*Cdc*20_ = 5, *θ*_*Cdc*14_ = 5, *θ*_*Plk*1_ = 5, *θ*_*Emi*1_ = 5, *θ*_*APC*_ = 5, *θ*_*E*2*F*_ = 5, *θ*_*CycE*_ = 5, *APC*_*tot*_ = 22, *Cdc*20_*tot*_ = 22, *Cdc*25_*tot*_ = 22, *Cdc*14_*tot*_ = 22.

### Fitzhugh-Nagumo model

We developed a relaxation oscillator model to characterize neuronal activity dynamics^29,33,34^. The model architecture is centered on two core Fitzhugh-Nagumo neurons that engage in mutual inhibition, forming a reciprocally inhibited pair capable of generating oscillatory dynamics. This central circuit is elaborated by supplementary regulatory connections. Each core neuron forms an additional positive feedback loop with one partner neuron and a negative feedback loop with another. These integrated feedback circuits modulate and fine-tune the oscillatory behaviors emerging from the core competitive interactions (Supplementary Fig. 2a).

11 $$\left\{\begin{array}{l}C{\dot{V}}_{i}={I}_{i}-F({V}_{i})-{W}_{i}\\ L{\dot{W}}_{i}=E-R{W}_{i}+{V}_{i}\end{array}\right.,i=1,2,\mathrm{..}.,N$$

12 $$F(V)=-V+{V}^{3}/3$$

13 $${I}_{i}={I}_{in}+\mathop{\sum }\limits_{j=1}^{N}{a}_{ij}S({V}_{j})$$

14 $$S(x)=\frac{1}{1+{e}^{-(x-{V}_{th})/{\theta }_{th}}}$$

Where *V* represents the voltage across the capacity, *W* represents the current through the resistor-inductor-battery circuit branch. *C* is the capacity, *R* is the resistance, *E* is the battery’s potential, *L* is the inductance, and *I*_*i*_ is the summed current to neuron *i*. *I*_*in*_ is the input current. *N* = 6 is the number of neurons, *a*_*ij*_ > 0 when neuron *j* excites neuron *i*. *a*_*ij*_ < 0 when neuron *j* inhibits neuron *i*. *a*_*ij*_ = 0 when neuron *j* doesn’t affect neuron *i*. *V*_*th*_ represents the threshold of the membrane potential. *θ*_*th*_ represents the time constant of the membrane potential.

Initial conditions:

All variables start with random values in [0,1].

Parameters:

*C* = 1, *L* = 12.5, *E* = 0.7, *R* = 0.8, *I*_*in*_ = 1, *a*_12_ = *k*_1_, *a*_21_ = *k*_2_, *a*_31_ = 1, *a*_42_ = 1, *a*_51_ = 0.9, *a*_62_ = 0.9, *a*_13_ = *neg*, *a*_24_ = *neg*, *a*_15_ = *pos*, *a*_26_ = *pos*.

### Goodwin oscillator model

We developed a Goodwin oscillator framework to model the *Arabidopsis thaliana* circadian clock, focusing on the core regulatory elements LHY and TOC1^35–39^. This core competitive interaction is extended through two primary negative feedback loops that define the oscillator’s dynamics. One where TOC1 protein represses GIGANTEA (GI) expression, and GI subsequently promotes TOC1 transcription, and another where LHY protein activates PSEUDO-RESPONSE REGULATOR 9 (PRR9) expression, and PRR9 then represses LHY transcription. The model is further elaborated by incorporating four supplementary regulatory proteins (LNK1, PRR5, PRR7, REV8) that form two additional positive feedback loops (LHY-LNK1 and TOC1-PRR5) and two additional negative feedback loops (LHY-PRR7 and TOC1-REV8) to fine-tune the oscillatory behaviors (Supplementary Fig. 2b). These modulatory elements act upon the core LHY/TOC1 module by regulating their synthesis or degradation rates, providing an additional layer of control consistent with the extended Goodwin oscillator paradigm.

15 $$\left\{\begin{array}{l}{\dot{X}}_{1}={v}_{1}\frac{1}{1+{{Z}_{1}}^{n}}-{v}_{2}{X}_{1}\\ {\dot{Y}}_{1}=({v}_{3}+{v}_{p}{W}_{1}){X}_{1}-({v}_{4}+{k}_{1}{Y}_{2}+{v}_{n}{U}_{1}){Y}_{1}\\ {\dot{Z}}_{1}={v}_{5}{Y}_{1}-{v}_{6}{Z}_{1}\\ {\dot{U}}_{1}={v}_{7}{Y}_{1}-{v}_{8}{U}_{1}\\ {\dot{W}}_{1}={v}_{9}{Y}_{1}-{v}_{10}{W}_{1}\\ {\dot{X}}_{2}={v}_{11}\frac{{{Z}_{2}}^{n}}{1+{{Z}_{2}}^{n}}-{v}_{12}{X}_{2}\\ {\dot{Y}}_{2}=\left({v}_{13}+{v}_{p}{W}_{2}+{v}_{21}{U}_{2}\right){X}_{2}-\left({v}_{14}+{k}_{2}{Y}_{1}\right){Y}_{2}\\ {\dot{Z}}_{2}={v}_{15}\frac{1}{1+{{Y}_{2}}^{n}}-{v}_{16}{Z}_{2}\\ {\dot{U}}_{2}={v}_{17}\frac{1}{1+{({v}_{n}{Y}_{2})}^{n}}-{v}_{18}{U}_{2}\\ {\dot{W}}_{2}={v}_{19}{Z}_{2}-{v}_{20}{W}_{2}\end{array}\right.$$

Where *X*_1_ represents the mRNA of LHY, *Y*_1_ represents the protein of LHY, *Z*_1_ represents the protein of PRR9, *U*_1_ represents the protein of PRR7, *W*_1_ represents the protein of LNK1, *X*_2_ represents the mRNA of TOC1, *Y*_2_ represents the protein of TOC1, *Z*_2_ represents the protein of GI, *U*_2_ represents the protein of RVE6, *W*_2_ represents the protein of PRR5.

Initial conditions:

All variables start with random values in [0,1].

Parameters:

*n* = 9, *v*_*n*_ = *neg*, *v*_*p*_ = *pos*, *v*_1_ = 1, *v*_2_ = 0.2, *v*_3_ = 1, *v*_4_ = 0.2, *v*_5_ = 1, *v*_6_ = 0.2, *v*_7_ = 0.3, *v*_8_ = 0.2, *v*_9_ = 0.3, *v*_10_ = 0.2, *v*_11_ = 0.2, *v*_12_ = 0.2, *v*_13_ = 1, *v*_14_ = 0.2, *v*_15_ = 0.2, *v*_16_ = 0.2, *v*_17_ = 0.3, *v*_18_ = 0.2, *v*_19_ = 0.3, *v*_20_ = 0.2, *v*_21_ = 0.3.

### Repressilator model

We developed a modified Repressilator system to engineer a complex genetic oscillator^40,41^. The core architecture consists of two distinct, interlinked Repressilator units, each a classic three-gene cyclic repression circuit, for a total of six core genes that provide the fundamental oscillatory behaviors. This core is augmented by four additional regulatory genes, which are integrated to form two positive and two negative feedback loops (Supplementary Fig. 2c). These supplementary circuits are designed to modulate and fine-tune the dynamics generated by the core Repressilators, resulting in a sophisticated ten-gene network.

16 $$\left\{\begin{array}{l}{\dot{X}}_{i}=\frac{1}{\tau }(-{X}_{i}+{H}_{i})\\ {\dot{Y}}_{i}=\frac{1}{\tau }\left({\alpha }_{i}{X}_{i}-{\beta }_{i}\left(1+{C}_{i}\right){Y}_{i}\right)\end{array}i=1,2{,}{.}{.}{.}{,}M\right.$$

17 $${H}_{i}=\left({v}_{0}+\mathop{\sum }\limits_{j=1}^{N}\frac{{({a}_{ij}{Y}_{j})}^{n}}{1+{({a}_{ij}{Y}_{j})}^{n}}\right)\cdot \mathop{\prod }\limits_{j=1}^{N}\frac{1}{1+{({b}_{ij}{Y}_{j})}^{n}}$$

18 $${C}_{i}=\mathop{\sum }\limits_{j=1}^{N}{c}_{ij}{Y}_{j}$$

Where *X* represents mRNA, and *Y* represents protein. *M* = 10 represents 10 groups of mRNA and proteins. *C*_*i*_ represents protein *j* enhances the degradation of protein *i*. *α* is the translation rate of mRNA into protein. *β* is the protein degradation rate. *v*_0_ is the basal mRNA synthesis rate. When *a*_*ij*_ > 0, gene *j* activates gene *i*. When *b*_*ij*_ > 0, gene *j* inhibits gene *i*. When *a*_*ij*_ = 0, *b*_*ij*_ = 0, there is no effect.

Initial conditions:

All variables start with random values in [0,1].

Parameters:

*τ* = 1, *n* = 5, *α* = 1, *β* = 0.1, *c*_14_ = *k*_1_, *c*_41_ = *k*_2_, *c*_17_ = *neg*, *c*_48_ = *neg*, *a*_19_ = *pos*, *a*_410_ = *pos*, *b*_21_ = 1, *b*_32_ = 1, *b*_13_ = 1, *b*_54_ = 1, *b*_65_ = 1, *b*_46_ = 1, *a*_71_ = 1, *a*_84_ = 1, *a*_91_ = 1, *a*_104_ = 1, *v*_0_ = 1.

### Predator-prey model

We developed a model of complex ecological interactions, grounded in the fundamental dynamic of interspecies competition^42–44^. The core framework consists of two predator species engaged in direct competition for two distinct prey populations, forming a system of mutually inhibited predators that generates intrinsic population oscillations. This competitive base is extended through regulatory mechanisms that incorporate additional species. Positive feedback loops are introduced to represent mutualistic symbionts, and negative feedback loops are implemented to simulate parasitic relationships (Supplementary Fig. 2d). The dynamics of this entire system are governed by the following coupled ODEs.

19 $$\left\{\begin{array}{l}{\dot{X}}_{1}=\alpha {X}_{1}(1-\frac{{X}_{1}}{K})-\beta \frac{{X}_{1}}{{X}_{1}+h}{Y}_{1}\\ {\dot{Y}}_{1}=\gamma \frac{{X}_{1}}{{X}_{1}+h}{Y}_{1}+{k}_{p}{Y}_{1}{Y}_{5}-{k}_{1}{Y}_{1}{Y}_{2}-{k}_{n}{Y}_{1}{Y}_{3}-\delta {Y}_{1}\\ {\dot{X}}_{2}=\alpha {X}_{2}\left(1-\frac{{X}_{2}}{K}\right)-\beta \frac{{X}_{2}}{{X}_{2}+h}{Y}_{2}\\ {\dot{Y}}_{2}=\gamma \frac{{X}_{2}}{{X}_{2}+h}{Y}_{2}+{k}_{p}{Y}_{2}{Y}_{6}-{k}_{2}{Y}_{1}{Y}_{2}-{k}_{n}{Y}_{2}{Y}_{4}-\delta {Y}_{2}\\ {\dot{Y}}_{3}=\alpha {Y}_{3}(1-\frac{{Y}_{3}}{K})+{\omega }_{1}{Y}_{1}{Y}_{3}-\delta {Y}_{3}\\ {\dot{Y}}_{4}=\alpha {Y}_{3}(1-\frac{{Y}_{3}}{K})+{\omega }_{1}{Y}_{2}{Y}_{4}-\delta {Y}_{4}\\ {\dot{Y}}_{5}=\alpha {Y}_{5}(1-\frac{{Y}_{5}}{K})+{\omega }_{2}{Y}_{1}{Y}_{5}-\delta {Y}_{5}\\ {\dot{Y}}_{6}=\alpha {Y}_{5}(1-\frac{{Y}_{5}}{K})+{\omega }_{2}{Y}_{2}{Y}_{6}-\delta {Y}_{6}\end{array}\right.$$

Where *X*_1_ and *X*_2_ denote the prey populations and *Y*_1_ and *Y*_2_ represent the predator populations. *Y*_3_ and *Y*_4_ represent the parasites. *Y*_5_ and *Y*_6_ represent the symbionts.

Initial conditions:

All variables start with random values in [0,1].

Parameters:

*α* = 1, *β* = 1, *h* = 0.5, *K* = 1, *γ* = 0.45, *δ* = 0.2, *ω*_1_ = 0.1, *ω*_2_ = 0.5, *k*_*n*_ = *neg*, *k*_*p*_ = *pos*.

### Van der Pol oscillator model

The competitive brain regions are modeled based on the Van der Pol model^10,45,46^. The competitive Van der Pol oscillators are mathematically described by: (i) a single-variable, second-order differential equation, (ii) an equivalent two-variable, first-order system formulation. We adapted this framework to model the two mutually inhibitory brain regions, PV1 and PV2. Each region’s activity is governed by the oscillator dynamics. Additional regulatory mechanisms implemented through positive feedback loops, [PV1 → SSTA1 → PV1] and [PV2 → SST2 → PV2], representing excitatory amplification, and negative feedback loops, [PV1 → PYR1 ⊣ PV1] and [PV2 → PYR2 ⊣ PV2], simulating inhibitory modulation (Supplementary Fig. 2e).

20 $$\left\{\begin{array}{l}{\dot{x}}_{i}=\frac{1}{\tau }({y}_{i}+{C}_{i})\\ {\dot{y}}_{i}=\frac{1}{\tau }(-{x}_{i}+\mu (1-{x}_{i}^{2}){y}_{i})\end{array}\right.$$

21 $${C}_{i}=\mathop{\sum }\limits_{j=1}^{N}{a}_{ij}{x}_{i}$$

*N* is the number of brain regions, *a*_*ij*_ > 0 when region *j* excites region *i*. *a*_*ij*_ < 0 when region *j* inhibits region *i*. *a*_*ij*_ = 0 when region *j* doesn’t affect region *i*.

Initial conditions:

All variables start with random values in [0,1].

Parameters:

*τ* = 3, *μ* = 0.1, *a*_12_ = *k*_1_, *a*_21_ = *k*_2_, *a*_13_ = *neg*, *a*_24_ = *neg*, *a*_15_ = *pos*, *a*_26_ = *pos*, *a*_31_ = 0.15, *a*_42_ = 0.15, *a*_51_ = 1, *a*_62_ = 0.1.

### Neuromechanical oscillator model

Informed by the structural constraints of musculature and their neural control^47,48^, we developed a neuromechanical model that integrates neurons and coupled muscle mechanics. The core component consists of two mutually inhibitory neuron-muscle feedback loops that generate oscillatory signals for movement. These circuits are abstracted into a simplified competitive architecture to capture essential biological dynamics. This neuronal network drives the mechanical system, where muscles function as the primary mutually inhibited elements due to anatomical constraints. Excitatory motor neurons induce muscle contraction, while inhibitory neurons promote relaxation. Muscle deformation is sensed by proprioceptive neurons, providing sensory feedback that closes the loop and sustains oscillation. The system is further modulated by supplementary pathways. Positive feedback loops arise from mechanical coupling between muscles, and additional negative feedback loops are formed through interactions between muscles and adjacent interneurons (Supplementary Fig. 2f). These regulatory circuits fine-tune the core oscillatory output. The dynamics of this coupled neuromechanical system are governed by the following system of ODEs.

22 $$\left\{\begin{array}{l}{\dot{x}}_{1}=(-{x}_{1}+{v}_{1}{u}_{neg11}+{v}_{2}{u}_{pos11}+{v}_{3}{u}_{gap13}+{u}_{in})/\tau \\ {\dot{y}}_{1}=(-{y}_{1}+{v}_{4}{u}_{ex11}+{u}_{ex13}+{u}_{inh16})/\tau \\ {\dot{z}}_{1}=(-{z}_{1}+{l}_{ex11}-{k}_{1}{l}_{inh12}-{k}_{p}{l}_{pos13})/\tau \\ {\dot{x}}_{2}=(-{x}_{2}+{v}_{1}{u}_{neg22}+{v}_{2}{u}_{pos22}+{v}_{3}{u}_{gap14}+{u}_{in})/\tau \\ {\dot{y}}_{2}=(-{y}_{2}+{u}_{ex22}+{u}_{ex24}+{u}_{inh25})/\tau \\ {\dot{z}}_{2}=(-{z}_{2}+{l}_{ex22}-{k}_{2}{l}_{inh21}-{k}_{p}{l}_{pos24})/\tau \\ {\dot{x}}_{3}=(-{x}_{3}+{k}_{n}{u}_{neg31}+{v}_{3}{u}_{gap31})/\tau \\ {\dot{x}}_{4}=(-{x}_{4}+{k}_{n}{u}_{neg42}+{v}_{3}{u}_{gap42})/\tau \\ {\dot{z}}_{3}=(-{z}_{3}-{v}_{5}{l}_{pos31})/\tau \\ {\dot{z}}_{4}=(-{z}_{4}-{v}_{5}{l}_{pos42})/\tau \\ {\dot{x}}_{5}=(-{x}_{5}+{u}_{ex51})/\tau \\ {\dot{x}}_{6}=(-{x}_{6}+{u}_{ex62})/\tau \end{array}\right.$$

23 $$\left\{\begin{array}{l}{u}_{neg11}=({E}_{inh}-{x}_{1})S({z}_{1},{N}_{th1})+({E}_{ex}-{x}_{1})(1-S({z}_{1},{N}_{th2}))\\ {u}_{pos11}=({E}_{ex}-{x}_{1})S({z}_{1},{P}_{th1})+({E}_{inh}-{x}_{1})(1-S({z}_{1},{P}_{th2}))\\ {u}_{gap13}={x}_{3}-{x}_{1}\\ {u}_{ex11}=({E}_{ex}-{y}_{1})S({x}_{1},{V}_{th})\\ {u}_{ex13}=({E}_{ex}-{y}_{1})S({x}_{3},{V}_{th})\\ {u}_{inh16}=({E}_{inh}-{y}_{1})S({x}_{6},{V}_{th})\\ {l}_{ex11}=({L}_{\max }-{z}_{1})S({y}_{1},{V}_{th})\\ {l}_{inh12}={z}_{2}S({z}_{2},{L}_{th})\\ {l}_{pos13}={z}_{3}\end{array}\right.$$

24 $$\left\{\begin{array}{l}{u}_{neg22}=({E}_{inh}-{x}_{2})S({z}_{2},{N}_{th1})+({E}_{ex}-{x}_{2})(1-S({z}_{2},{N}_{th2}))\\ {u}_{pos22}=({E}_{ex}-{x}_{2})S({z}_{2},{P}_{th1})+({E}_{inh}-{x}_{2})(1-S({z}_{2},{P}_{th2}))\\ {u}_{gap24}={x}_{4}-{x}_{2}\\ {u}_{ex22}=({E}_{ex}-{y}_{2})S({x}_{2},{V}_{th})\\ {u}_{ex24}=({E}_{ex}-{y}_{2})S({x}_{4},{V}_{th})\\ {u}_{inh25}=({E}_{inh}-{y}_{2})S({x}_{5},{V}_{th})\\ {l}_{ex22}=({L}_{\max }-{z}_{2})S({y}_{2},{V}_{th})\\ {l}_{inh21}={z}_{1}S({z}_{1},{L}_{th})\\ {l}_{pos24}={z}_{4}\\ {u}_{neg31}=({E}_{inh}-{x}_{3})S({z}_{1},{N}_{th1})+({E}_{ex}-{x}_{3})(1-S({z}_{1},{N}_{th2}))\\ {u}_{gap31}={x}_{1}-{x}_{3}\end{array}\right.$$

25 $$\left\{\begin{array}{l}{u}_{neg42}=({E}_{inh}-{x}_{4})S({z}_{2},{N}_{th1})+({E}_{ex}-{x}_{4})(1-S({z}_{2},{N}_{th2}))\\ {u}_{gap42}={x}_{2}-{x}_{4}\\ {l}_{pos31}={z}_{1}\\ {l}_{pos42}={z}_{2}\\ {u}_{ex51}=({E}_{ex}-{x}_{5})S({x}_{1},{V}_{th})\\ {u}_{ex62}=({E}_{ex}-{x}_{6})S({x}_{2},{V}_{th})\end{array}\right.$$

26 $$S(x,{X}_{th})=\frac{1}{1+{e}^{({X}_{th}-x)/0.01}}$$

Where *x* represents the membrane of a neuron, *y* represents the membrane of a muscle, and *z* represents the deformation of the muscle.

Initial conditions:

All variables start with random values in [0,1].

Parameters:

*τ* = 0.25, *v*_1_ = 20, *v*_2_ = 0.2, *v*_3_ = 1, *v*_4_ = 2, *v*_5_ = 0.5, *u*_*in*_ = 1, *E*_*ex*_ = 2, *E*_*inh*_ = −2, *L*_*max*_ = 2, *k*_*n*_ = *neg*, *k*_*p*_ = *pos*, *N*_*th*1_ = 0.5, *N*_*th*2_ = −0.5, *P*_*th*1_ = 0.1, *P*_*th*2_ = −0.1, *L*_*th*_ = 0.01, *V*_*th*_ = 0.5.

## Supplementary information

Supplementary information

Supplementary Data 1

## Data Availability

Source data are provided in this paper as Supplementary Data 1. All data are also available by request to the corresponding authors.

## Code availability

The source code used in this work is available 10.5281/zenodo.16910330.

## References

[1] Huikuri, H. & Mäkikallio, T. Heart rate variability in ischemic heart disease. *Auton. Neurosci.* **90**, 95–101 (2001). 11485298 10.1016/S1566-0702(01)00273-9

[2] Asai, Y. et al. A coupled oscillator model of disordered interlimb coordination in patients with Parkinson’s disease. *Biol. Cybern.* **88**, 152–162 (2003). 12567229 10.1007/s00422-002-0371-9

[3] Uhlhaas, P. & Singer, W. Abnormal neural oscillations and synchrony in schizophrenia. *Nat. Rev. Neurosci.* **11**, 100–113 (2010). 20087360 10.1038/nrn2774

[4] Gery, S. & Koeffler, H. Circadian rhythms and cancer. *Cell Cycle* **9**, 1097–1103 (2010). 20237421 10.4161/cc.9.6.11046 PMC5884684

[5] Rijo-Ferreira, F. & Takahashi, J. Genomics of circadian rhythms in health and disease. *Genome Med.* **11**, 82 (2019). 31847894 10.1186/s13073-019-0704-0 PMC6916512

[6] Ferrell, J. E., Tsai, T. Y. & Yang, Q. Modeling the cell cycle: why do certain circuits oscillate?. *Cell* **144**, 874–885 (2011). 21414480 10.1016/j.cell.2011.03.006

[7] Liu, J. et al. Coupling between distant biofilms and emergence of nutrient time-sharing. *Science* **356**, 638–641 (2017). 28386026 10.1126/science.aah4204 PMC5645014

[8] Wang, L. et al. Modulation of dynamic modes by interplay between positive and negative feedback loops in gene regulatory networks. *Phys. Rev. E* **97**, 042412 (2018). 29758769 10.1103/PhysRevE.97.042412

[9] Loveless, J., Lagogiannis, K. & Webb, B. Modelling the mechanics of exploration in larval Drosophila. *PLoS Comput. Biol.* **15**, e1006635 (2019). 31276489 10.1371/journal.pcbi.1006635 PMC6636753

[10] Hahn, G., Kumar, A., Schmidt, H., Knoesche, T. & Deco, G. Rate and oscillatory switching dynamics of a multilayer visual microcircuit model. *eLife* **11**, e77594 (2022). 35994330 10.7554/eLife.77594 PMC9395191

[11] Kim, H., Yoo, T., Cho, J., Kim, H. & Lee, W. Indoxyl sulfate-induced TNF-α is regulated by crosstalk between the aryl hydrocarbon receptor, NF-κB, and SOCS2 in human macrophages. *FASEB J.* **33**, 10844–10858 (2019). 31284759 10.1096/fj.201900730R

[12] Tong, Y. et al. DPP3/CDK1 contributes to the progression of colorectal cancer through regulating cell proliferation, cell apoptosis, and cell migration. *Cell Death Dis.* **12**, 623 (2021). 34135316 10.1038/s41419-021-03886-3 PMC8209024

[13] Göttlich, M. et al. Altered resting state brain networks in Parkinson’s disease. *PLoS One* **8**, e77336 (2013). 24204812 10.1371/journal.pone.0077336 PMC3810472

[14] Scammell, T., Arrigoni, E. & Lipton, J. Neural circuitry of wakefulness and sleep. *Neuron* **93**, 747–765 (2017). 28231463 10.1016/j.neuron.2017.01.014 PMC5325713

[15] Liu, A. C. et al. Redundant function of REV-ERBα and β and non-essential role for BMAL1 cycling in transcriptional regulation of intracellular circadian rhythms. *PLoS Genet* **4**, e1000023 (2008). 18454201 10.1371/journal.pgen.1000023 PMC2265523

[16] Kim, J., Shin, D., Jung, S., Heslop-Harrison, P. & Cho, K. A design principle underlying the synchronization of oscillations in cellular systems. *J. Cell Sci.* **123**, 537–543 (2010). 20103537 10.1242/jcs.060061

[17] Lahav, G. et al. Dynamics of the p53-Mdm2 feedback loop in individual cells. *Nat. Genet.* **36**, 147–150 (2004). 14730303 10.1038/ng1293

[18] Novák, B. & Tyson, J. Design principles of biochemical oscillators. *Nat. Rev. Mol. Cell Biol.* **9**, 981–991 (2008). 18971947 10.1038/nrm2530 PMC2796343

[19] Li, Z., Liu, S. & Yang, Q. Incoherent inputs enhance the robustness of biological oscillators. *Cell Syst.* **5**, 72–81 (2017). 28750200 10.1016/j.cels.2017.06.013 PMC5564455

[20] Tigges, M., Marquez-Lago, T. T., Stelling, J. & Fussenegger, M. A tunable synthetic mammalian oscillator. *Nature* **457**, 309–312 (2009). 19148099 10.1038/nature07616

[21] Cao, Y., Lopatkin, A. & You, L. Elements of biological oscillations in time and space. *Nat. Struct. Mol. Biol.* **23**, 1030–1034 (2016). 27922613 10.1038/nsmb.3320

[22] Tsai, T. Y. C. et al. Robust, tunable biological oscillations from interlinked positive and negative feedback loops. *Science* **321**, 126–129 (2008). 18599789 10.1126/science.1156951 PMC2728800

[23] Ferrell, J. Feedback loops and reciprocal regulation: recurring motifs in the systems biology of the cell cycle. *Curr. Opin. Cell Biol.* **25**, 676–686 (2013). 23927869 10.1016/j.ceb.2013.07.007 PMC3836843

[24] Wang, G., Yang, Z. & Turcotte, M. Dynamic analysis of the time-delayed genetic regulatory network between two auto-regulated and mutually inhibitory genes. *Bull. Math. Biol.* **82**, 46 (2020). 32236721 10.1007/s11538-020-00722-1

[25] Murugan, R. & Kreiman, G. Multiple transcription auto regulatory loops can act as robust oscillators and decision-making motifs. *Comp. Struct. Biotechnol. J.* **20**, 5115–5135 (2022). 10.1016/j.csbj.2022.08.065 PMC9493064 36187915

[26] Szymanska, P., Martin, K., MacKeigan, J., Hlavacek, W. & Lipniacki, T. Computational analysis of an autophagy/translation switch based on mutual inhibition of MTORC1 and ULK1. *PLoS ONE* **10**, e0116550 (2015). 25761126 10.1371/journal.pone.0116550 PMC4356596

[27] Keeley, S., Fenton, A. & Rinzel, J. Modeling fast and slow gamma oscillations with interneurons of different subtype. *J. Neurophysiol.* **117**, 950–965 (2017). 27927782 10.1152/jn.00490.2016 PMC5338627

[28] Tian, X., Zhang, X., Liu, F. & Wang, W. Interlinking positive and negative feedback loops creates a tunable motif in gene regulatory networks. *Phys. Rev. E* **80**, 011926 (2009). 10.1103/PhysRevE.80.011926 19658748

[29] Labavic, D. & Meyer-Ortmanns, H. Networks of coupled circuits: from a versatile toggle switch to collective coherent behavior. *Chaos* **24**, 043118 (2014). 25554038 10.1063/1.4898795

[30] Pett, J., Korencic, A., Wesener, F., Kramer, A. & Herzel, H. Feedback loops of the mammalian circadian clock constitute repressilator. *PLoS Comput. Biol.* **12**, e1005266 (2016). 27942033 10.1371/journal.pcbi.1005266 PMC5189953

[31] Zeng, Y. & Wiens, J. Species interactions have predictable impacts on diversification. *Ecol. Lett.* **24**, 239–248 (2021). 33146947 10.1111/ele.13635

[32] Dragoi, C., Kaur, E., Barr, A., Tyson, J. & Novák, B. The oscillation of mitotic kinase governs cell cycle latches in mammalian cells. *J. Cell Sci.* **137**, jcs261364 (2024). 38206091 10.1242/jcs.261364 PMC10911285

[33] FitzHugh, R. Mathematical models of threshold phenomena in the nerve membrane. *Bull. Math. Biophys.* **17**, 257–278 (1955).

[34] Nagumo, J., Arimoto, S. & Yoshizawa, S. An active pulse transmission line simulating nerve axon. *Proc. IRE* **50**, 2061–2070 (1962).

[35] Goodwin, B. C. Oscillatory behavior in enzymatic control processes. *Adv. Enzym. Regul.* **3**, 425–438 (1965). 10.1016/0065-2571(65)90067-1 5861813

[36] Ruoff, P., Mohsenzadeh, S. & Rensing, L. Circadian rhythms and protein turnover: the effect of temperature on the period lengths of clock mutants simulated by the Goodwin oscillator. *Naturwissenschaften* **83**, 514–517 (1996). 8971726 10.1007/BF01141953

[37] Smolen, P., Baxter, D. & Byrne, J. A reduced model clarifies the role of feedback loops and time delays in the Drosophila circadian oscillator. *Biophys. J.* **83**, 2349–2359 (2002). 12414672 10.1016/S0006-3495(02)75249-1 PMC1302324

[38] Du, S., Chen, L., Ge, L. & Huang, W. A novel loop: mutual regulation between epigenetic modification and the circadian clock. *Front. Plant Sci.* **10**, 22 (2019). 30761168 10.3389/fpls.2019.00022 PMC6362098

[39] Saini, R., Jaskolski, M. & Davis, S. Circadian oscillator proteins across the kingdoms of life: structural aspects. *BMC Biol.* **17**, 13 (2019). 30777051 10.1186/s12915-018-0623-3 PMC6378743

[40] Elowitz, M. & Leibler, S. A synthetic oscillatory network of transcriptional regulators. *Nature* **403**, 335–338 (2000). 10659856 10.1038/35002125

[41] Gonze, D. Coupling oscillations and switches in genetic networks. *Biosystems* **99**, 60–69 (2010). 19735694 10.1016/j.biosystems.2009.08.009

[42] Yoshida, T., Jones, L., Ellner, S., Fussmann, G. & Hairston, N. Rapid evolution drives ecological dynamics in a predator-prey system. *Nature* **424**, 303–306 (2003). 12867979 10.1038/nature01767

[43] Bate, A. & Hilker, F. Predator-prey oscillations can shift when diseases become endemic. *J. Theor. Biol.* **316**, 1–8 (2013). 23017445 10.1016/j.jtbi.2012.09.013

[44] Colon, C., Claessen, D. & Ghil, M. Bifurcation analysis of an agent-based model for predator-prey interactions. *Ecol. Model.* **317**, 93–106 (2015).

[45] Yamapi, R., Filatrella, G. & Aziz-Alaoui, M. A. Global stability analysis of birhythmicity in a self-sustained oscillator. *Chaos* **20**, 013114 (2010). 20370269 10.1063/1.3309014

[46] Petkoski, S. & Jirsa, V. K. Transmission time delays organize the brain network synchronization. *Philos. Trans. R. Soc. A Math. Phys. Eng. Sci.* **377**, 20180132 (2019). 10.1098/rsta.2018.0132 PMC6661323 31329065

[47] Boyle, J. H., Berri, S. & Cohen, N. Gait modulation in C. elegans: an integrated neuromechanical model. *Front. Comput. Neurosci.* **6**, 10 (2012). 22408616 10.3389/fncom.2012.00010 PMC3296079

[48] Olivares, E. O., Izquierdo, E. J. & Beer, R. D. Potential role of a ventral nerve cord central pattern generator in forward and backward locomotion in Caenorhabditis elegans. *Netw. Neurosci.* **2**, 323–343 (2018). 30294702 10.1162/netn_a_00036 PMC6145852

