# Supplementary material for: Flexible modulation of hybrid feedback loops in competitive biological oscillators
Source: NPJ Syst Biol Appl. 2025 Nov 3;11:122. doi: 10.1038/s41540-025-00594-y (PMC12583529; doi:10.1038/s41540-025-00594-y)
Supplement: Supplementary file 1 — Supplementary information [file 41540_2025_594_MOESM1_ESM.pdf]

Supplementary Information for

Flexible modulation of hybrid feedback loops in competitive biological oscillators

Peng Zhao, Jian Liu, Tengfei Bao, Hong Huo, Ye Yuan, Tao Fang

Corresponding author: Ye Yuan, yuanye_usst@usst.edu.cn; Tao Fang, tfang@sjtu.edu.cn

The PDF file includes:

Supplementary Figure 1. Modulation of Cdh1 oscillation properties by feedback regulation
Supplementary Figure 2. Hybrid feedback architecture across biological oscillator models
Supplementary Figure 3. Phase portraits and time series across biological oscillator models
Supplementary Figure 4. Amplitude and frequency as functions of feedback strength across biological oscillator models
Supplementary Data 1. Source data for all main and supplementary figures

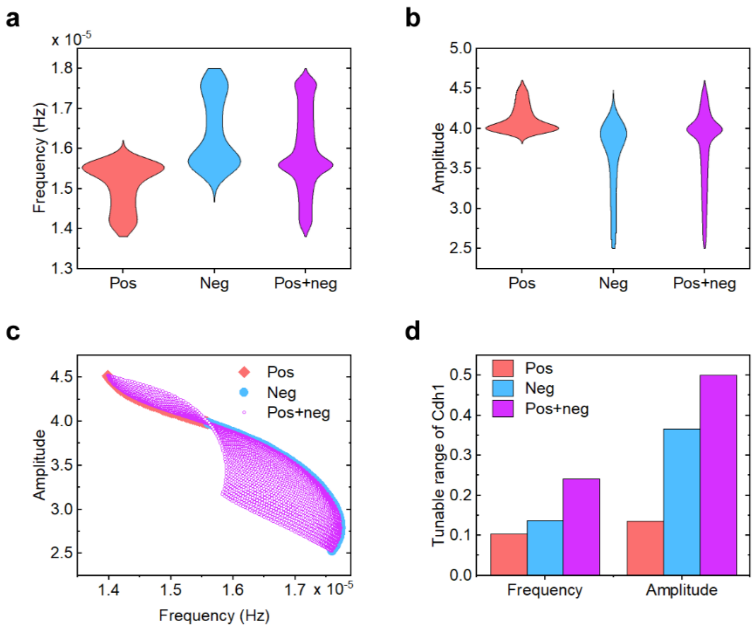

Supplementary Fig. 1 | Modulation of Cdh1 oscillation properties by feedback regulation. a Frequency distributions under three feedback conditions: positive feedback only, negative feedback only, and hybrid feedback. **b** Corresponding amplitude distributions, with violin plot width representing probability density. **c** The amplitude/frequency scatters across all feedback conditions. **d** Normalized tunability ranges for Cdh1 frequency (left) and amplitude (right) under each feedback configuration. Tunability was calculated as (maximum - minimum)/reference value, where reference value represents the no-feedback baseline condition (*pos* = 0, *neg* = 0).

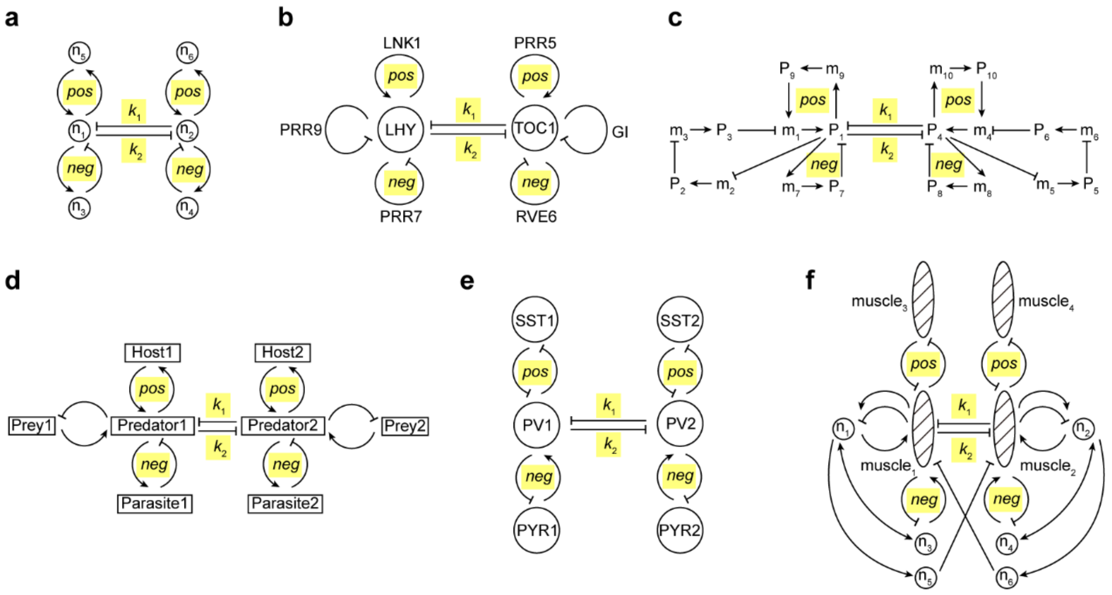

Supplementary Fig. 2 | Hybrid feedback architecture across biological oscillator models. Core competitive structures with regulatory feedback loops in six biological models: **a** Fitzhugh-Nagumo model in neuronal networks. **b** Goodwin model for circadian rhythms. **c** Repressilator model in gene regulatory networks. **d** Predator-prey model in the ecological system. **e** Van der Pol for brain region activity. **f** Neuromechanical oscillator for motor control. All models include: (i) core competitive interactions between principal components, (ii) additional regulatory circuits composed of positive feedback and negative feedback loops.

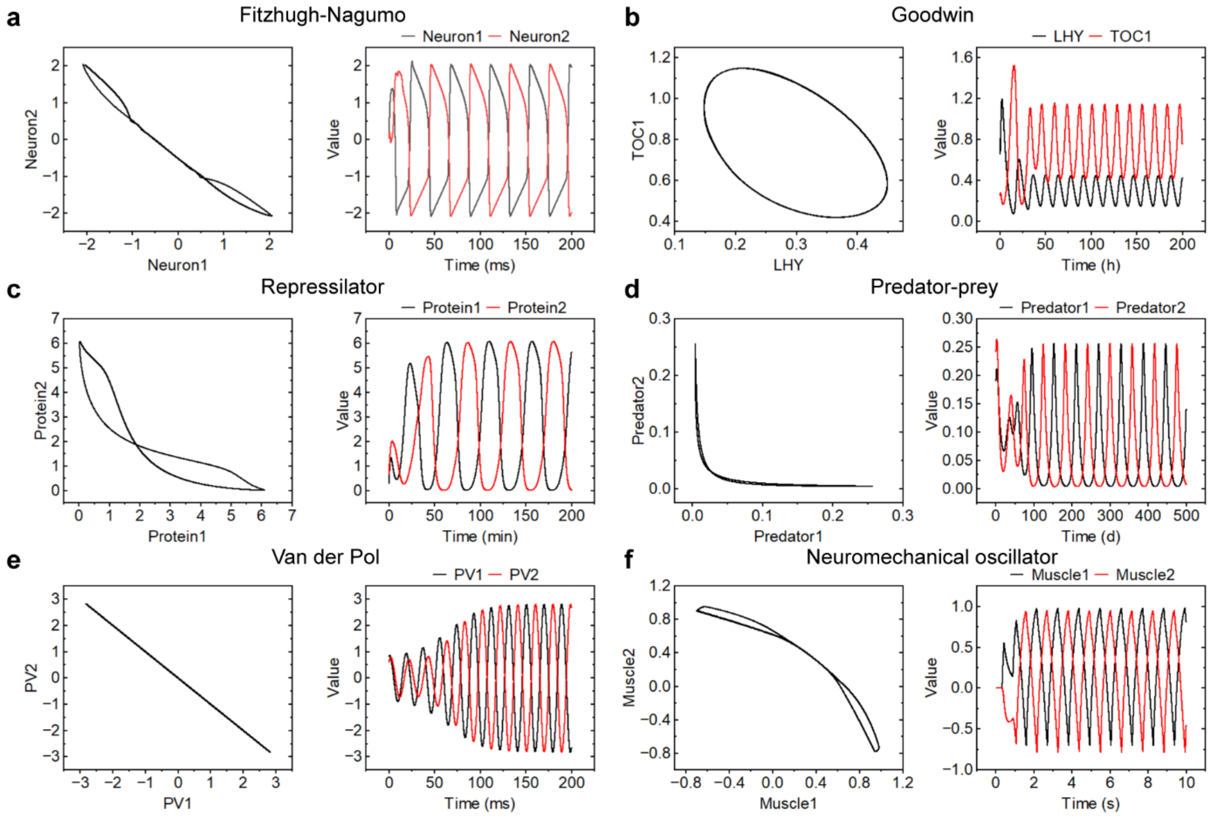

Supplementary Fig. 3 | Phase portraits and time series across biological oscillator models. The phase portraits (left) and time series (right) of key variables in six biological models: **a** Fitzhugh-Nagumo model. **b** Goodwin model. **c** Repressilator model. **d** Predator-prey model. **e** Van der Pol model. **f** Neuromechanical oscillator model.

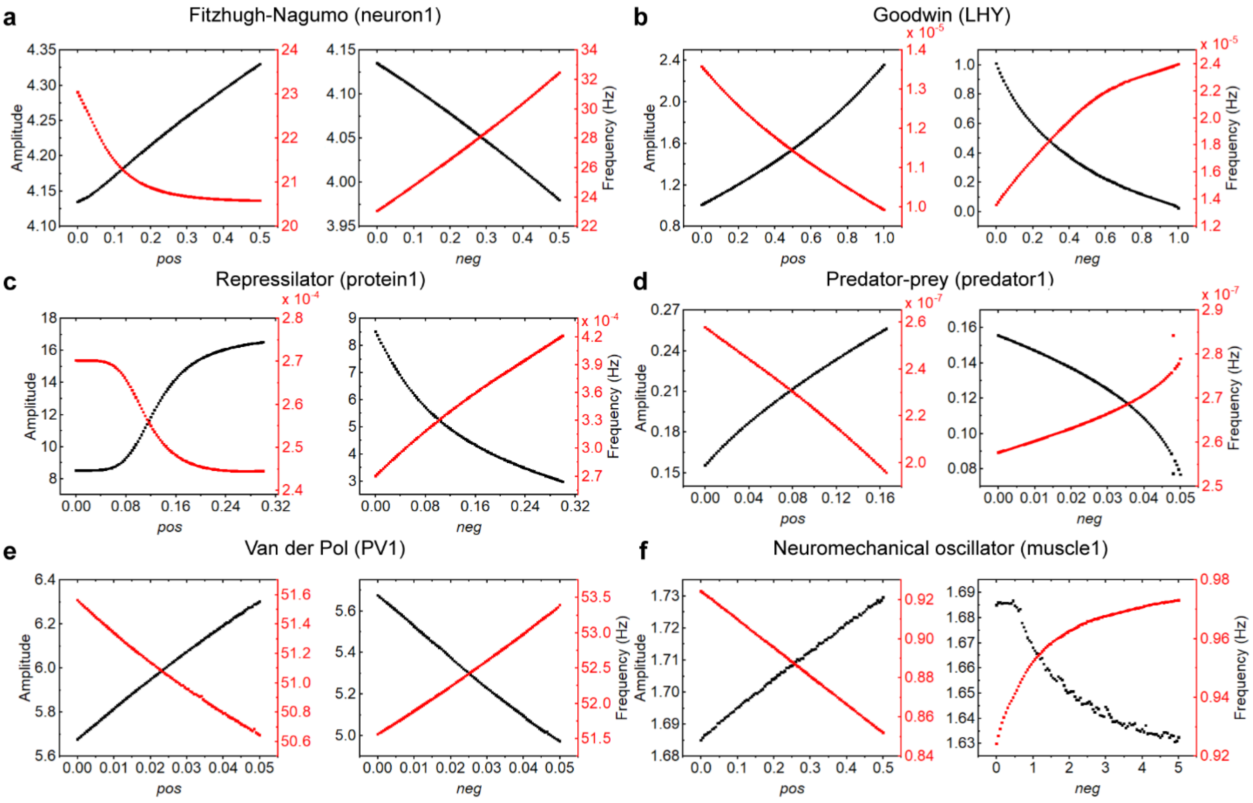

Supplementary Fig. 4 | Amplitude and frequency as functions of feedback strength across biological oscillator models. Amplitude and frequency as functions of positive feedback strength (left) and negative feedback strength (right) of one key variable in six biological models: **a** Fitzhugh-Nagumo model (neuron1). **b** Goodwin model (LHY). **c** Repressilator model (protein1). **d** Predator-prey model (predator1). **e** Van der Pol model (PV1). **f** Neuromechanical oscillator model (muscle1).

Supplementary Data 1 (legend). **Source data for all main and supplementary figures**. We provide the raw data used to plot all graphs, presented in the main article Figures 1-5 and Supplementary Figures 1-4. Data are organized by figure panel in separate spreadsheets.
